# Molecular Investigation of the Effects of Two Antiepileptic Drugs (Valproic Acid and Levetiracetam) on Alveolar Bone Under Orthodontic Force

**DOI:** 10.3390/medicina62010178

**Published:** 2026-01-15

**Authors:** Nurhan Bayindir-Durna, Metin Uckan, Seyma Aydin, Selcuk Ozdemir

**Affiliations:** 1 Faculty of Dentistry, Atatürk University, Erzurum 25240, Turkey; metinuckann@gmail.com; 2 Department of Genetics, Faculty of Veterinary Medicine, Atatürk University, Erzurum 25240, Turkey; seyma.aydin@atauni.edu.tr (S.A.); selcuk.ozdemir@atauni.edu.tr (S.O.)

**Keywords:** apoptosis, inflammation, bone resorption, levetiracetam, oxidative stress, valproic acid, antiepileptic drugs

## Abstract

*Background and Objectives:* This study aims to analyze the effects of levetiracetam (LEV) and valproic acid (VPA) administration on oxidative stress, inflammation, apoptosis, extracellular matrix dynamics, and bone remodeling parameters in rat alveolar bone exposed to orthodontic force. *Materials and Methods:* Four experimental groups were designed for this study: Control, Force, Force + LEV, and Force + VPA. LEV (150 mg/kg/day) or VPA (300 mg/kg/day) was administered intraperitoneally to the experimental groups daily for 6 weeks. At the end of the experimental period, the alveolar bone tissues were used for molecular analyses. RT-PCR analysis was performed to assess the expression levels of antioxidant markers [superoxide dismutase, (SOD), catalase (CAT), glutathione peroxidase (GPx), and glutathione (GSH)], inflammatory cytokines [tumor necrosis factor alpha (TNF-α) and interleukin-1 beta (IL-1β)], apoptosis-related genes (Bax, Bcl-2, and Caspase-3), matrix remodeling genes [matrix metalloproteinase-2 (MMP-2), matrix metalloproteinase-9 (MMP-9), and metallopeptidase inhibitor 1 (TIMP-1)], and bone metabolism regulators [receptor activator of nuclear factor kappa-Β ligand (RANKL) and osteoprotegerin (OPG)]. Oxidative stress and inflammatory measurements were also confirmed via ELISA assays. *Results:* The results demonstrated that orthodontic force application increased oxidative stress, inflammation, and apoptosis compared to the Control group, disrupted extracellular matrix homeostasis, and increased bone resorption, while LEV administration (LEV + Force) markedly mitigated these abnormalities. In other words, LEV administration increased levels of antioxidant markers, decreased levels of inflammatory cytokines and pro-apoptotic genes, restored extracellular matrix balance (decrease in MMP-2 and MMP-9 with concurrent upregulation of TIMP-1), and limited tissue destruction (decrease in RANKL along with elevation in OPG). In contrast to LEV, VPA did not correct these molecular alterations induced by orthodontic force and, in several parameters, further exacerbated them. *Conclusions:* In conclusion, molecular data from the animal model indicate that LEV plays a protective role against orthodontic force by reducing excess levels of oxidative stress, apoptosis, and inflammation and homeostatic pathways.

## 1. Introduction

In dentistry, orthodontic force is mainly used to move teeth, thereby correcting malocclusion (jawbone misalignment resulting from the incorrect and improper alignment of teeth), guiding jaw and facial development (especially in children and adolescents), and improving chewing and speech functions [1,2,3]. Orthodontic force, a controlled mechanical force applied to a tooth, causes biologic changes and triggers cell signaling cascades in the periodontal tissue (ligament) and alveolar bone, thereby enabling tissue remodeling and tooth movement [4]. The movement of the tooth within the alveolar bone is essentially a physiological response of the periodontal tissue to mechanical stimuli applied to the tooth [5]. Under pressure or tension, the periodontal tissue and alveolar bone undergo osteoclastic and osteogenic activities, respectively, resulting in tooth displacement. Therefore, the rate of tooth movement and the risk of root resorption are largely determined by how mechanical forces are perceived, transmitted, and responded to at the cellular and tissue levels [5,6].

With respect to pressure, orthodontic force causes the compression of periodontal tissue, a reduction in blood flow, the stimulation of osteoclasts, and the enhancement of bone resorption. This force also stretches periodontal tissue, enhances blood flow, stimulates osteoblasts, and causes new bone formation on the tension side [6,7]. Orthodontic force affects tooth movement and tissue remodeling through different cellular and molecular mechanisms, such as inflammation, oxidative stress, and apoptosis [8,9,10]. For example, it is known that the normal level of orthodontic force-induced inflammation is essential for osteoclast activation and tooth movement, as well as bone resorption on the pressure side. Similarly, oxidative stress at normal levels is known to be beneficial, since it enhances osteoclast differentiation and supports normal bone remodeling by acting as a cell signaling mechanism. However, excess oxidative stress and inflammation cause tissue damage and prevent normal bone remodeling and tooth movement [8,11,12,13]. Therefore, oxidative stress, inflammation, and apoptosis levels need to be controlled in patients under orthodontic force.

Valproic acid (VPA) and levetiracetam (LEV) are second-line anti-seizure drugs that are widely used to treat epilepsy. VPA is a broad-spectrum antiepileptic drug, while LEV is a newer (second-generation) antiepileptic drug. Both antiepileptic drugs, especially VPA, are known to cause unwanted adverse effects in humans [14,15,16,17]. In addition, it has been shown that the two drugs exhibit some adverse effects in dentistry. For example, it has been reported that VPA increases the bleeding risk and causes gingival changes in teeth [18,19]. LEV can induce gingival hyperplasia or gingival enlargement in humans [20,21]. Some studies have also focused on the effect of these two drugs on bone and mineral density. However, those studies analyzed changes in other bone tissues other than teeth [22,23]. For instance, Xie and colleagues [23] demonstrated that VPA reduced bone mass and bone mineral density at lumbar spine 1, the femoral neck, and total hip in both patients and mice; however, their study did not directly focus on the effects of VPA on bone mass and bone mineral density in the tooth. A prospective study [22] elucidated that there was no significant reduction in the bone mineral density of the lumbar spine of people with epilepsy treated with LEV. In summary, it can be concluded that there is no direct evidence of the effects of VPA or LEV on periodontal tissues and alveolar bone exposed to orthodontic force. Furthermore, no study in the literature has examined the effects of VPA and LEV on the molecular mechanisms (oxidative stress, inflammation, and apoptosis) that regulate the bone remodeling process.

Accordingly, this study was conducted to determine the effects of antiepileptic drugs (VPA and LEV) on the bone remodeling mechanism of alveolar bone exposed to orthodontic force in a rat model. The aim is to obtain preliminary findings regarding which of these medications (VPA or LEV) should be preferred in epileptic patients who require orthodontic treatment. In this study, the effects of VPA and LEV on alveolar bone were evaluated in the context of oxidative stress, inflammation, apoptosis, matrix remodeling, and bone remodeling mechanisms.

## 2. Materials and Methods

### 2.1. Animal Experiments and Study Design

This study used 9–10-week-old Wistar albino male rats, weighing 250–300 g, obtained from ATADEM (Atatürk University Medical Experimental Application and Research Center, Erzurum, Türkiye). Male rats were preferred in this study, as hormonal changes in female rats due to their menstrual cycles could affect the experimental results.

At the beginning of this study, 40 Wistar albino male rats were randomly divided into 4 groups, with 10 rats in each group:

1. Control Group (Negative Control Group): After completing a 1-week acclimation period, the rats in this group were administered only saline solution intraperitoneally throughout the experimental period. No medication was administered, and no force was applied.
2. Force Group (Operated Control Group): In this group of rats, following a one-week acclimatization period, a nickel–titanium closed-coil spring was placed between the maxillary left first molar and the incisor to apply orthodontic force. The spring was fixed in position to provide a continuous force and remained in the oral cavity for six weeks without reactivation. Starting from the day of appliance placement, the rats received daily intraperitoneal saline solution injections, administered at the same time each day throughout the 6-week experimental period.
3. Force + Valproic Acid (VPA) Group: In this group of rats, following a one-week acclimatization period, a nickel–titanium closed-coil spring was placed between the maxillary left first molar and the incisor to apply orthodontic force. The spring was fixed in position to provide a continuous force and remained in the oral cavity for six weeks without reactivation. Starting from the day of appliance placement, the rats received daily intraperitoneal injections of valproic acid at a dose of 300 mg/kg, administered at the same time each day throughout the 6-week experimental period.
4. Force + Levetiracetam (LEV) Group: In this group of rats, following a one-week acclimatization period, a nickel–titanium closed-coil spring was placed between the maxillary left first molar and the incisor to apply orthodontic force. The spring was fixed in position to provide a continuous force and remained in the oral cavity for six weeks without reactivation. Starting from the day of appliance placement, the rats received daily intraperitoneal injections of levetiracetam at a dose of 150 mg/kg, administered at the same time each day throughout the 6-week experimental period.

All rats were housed in polycarbonate cages at 22 ± 2 °C, with 12 h of light and 12 h of darkness. Standard pellet feed and tap water were used for feeding. The rats’ feed and water consumption, general health, and appliance retention were monitored daily, and the removed appliances were replaced the same day. The animals’ body weights were measured at the beginning of the experiment and weekly. A 15% weight loss during the experiment was established as an exclusion criterion. All animals completed the experiment without significant health problems and with full appliance retention.

**Figure 1:**
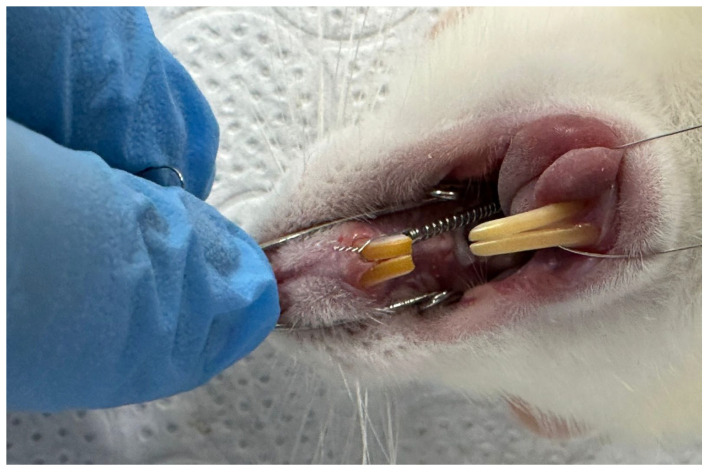
Orthodontic tooth movement in rats using a NiTi closed-coil spring.

Rats scheduled to receive orthodontic force after a one-week adaptation period were anesthetized via the intramuscular administration of a combination of 5–13 mg/kg xylazine hydrochloride and 75–100 mg/kg ketamine hydrochloride, depending on their weight [24]. Orthodontic tooth movement was achieved using a 6 mm long NiTi closed helical spring (COCODENT, Shenzhen, China; Cat No: 07CS10060000) placed between the left first molars and incisors of the upper jaw to apply continuous force for 6 weeks (Figure 1). A 0.010 mm stainless-steel ligature wire was placed in the interproximal space of the maxillary left first and second molars of the rats and fixed to the mesial of the left first molar. Then, retention grooves were opened at the gingival level on the mesial, distal, and buccal surfaces of the incisors with a flame-tipped hard bur, and a second ligature wire was passed through the prepared grooves. Using an intraoral force gauge, a NiTi closed-coil spring was tensioned to apply 50 g of force and connected to the ligature wire on the incisors. The amount of force applied in our study was determined using references from the literature [25,26,27].

In the literature, a dose range of 150–300 mg/kg/day has generally been preferred for chronic VPA administration in rat models [28,29]. Similarly, in rat studies using levetiracetam, doses in the range of 50–150 mg/kg/day have been reported to be safe and sufficient for evaluating effects on bone tissue [30,31,32]. Therefore, in the case of our study, based on human equivalent doses, doses of 150 mg/kg/day for LEV and 300 mg/kg/day for VPA were preferred.

At the end of the experimental period, the animals were sacrificed by administering a high-dose anesthetic solution. Following sacrifice, the rats’ heads were separated from their bodies, and the maxillae were dissected from the incisors to the soft palate using surgical scissors. The resulting tissue samples were preserved under appropriate conditions for the planned histological and molecular analyses.

### 2.2. Analysis of Oxidative Stress and Inflammation Markers

Analysis of oxidative stress and inflammation markers was performed on bone tissue samples obtained from the experimental groups (Control, Force, Force + LEV, and Force + VPA). Antioxidant marker levels were normalized to total protein content and expressed per milligram of protein. The total protein concentration was determined using the Bradford protein assay (Bradford Reagent, Sigma-Aldrich, St. Louis, MO, USA; Cat. No: B6916), according to the manufacturer’s instructions.

Oxidative stress status was evaluated by measuring the activities of antioxidant enzymes, including superoxide dismutase (SOD), catalase (CAT), and glutathione peroxidase (GPx), as well as reduced glutathione (GSH) levels. These parameters were quantified in strict accordance with the manufacturers’ protocols using commercially available ELISA kits: SOD (MyBioSource, San Diego, CA, USA; Cat. No: MBS036924), CAT (MyBioSource, USA; Cat. No: MBS2600683), GSH (MyBioSource, USA; Cat. No: MBS9712516), and GPx (MyBioSource, USA; Cat. No: MBS744364).

Inflammatory responses were assessed by determining the levels of the pro-inflammatory cytokines tumor necrosis factor-alpha (TNF-α) and interleukin-1 beta (IL-1β) using rat-specific ELISA kits (MyBioSource, USA; Cat. No: MBS282960 and MBS265868, respectively).

All samples were analyzed in duplicate. Absorbance values were measured using a microplate reader (BioTek Instruments, Winooski, VT, USA; Model: ELx800) at assay-specific wavelengths recommended by the manufacturers.

### 2.3. Gene Expression Analysis

#### 2.3.1. Total RNA Isolation

Bone tissue samples were immediately cryopreserved in liquid nitrogen and subsequently homogenized using a Qiagen TissueLyser 3 system (Qiagen, Hilden, Germany). Approximately 50 mg of bone tissue from each experimental group was homogenized in 1 mL of Qiazol reagent (QIAzol Lysis Reagent, Qiagen, Hilden, Germany; Cat. No: 79306) and incubated for 5 min at room temperature to ensure complete dissociation of nucleoprotein complexes.

Following centrifugation at 12,000× *g* for 15 min at 4 °C, the aqueous phase was carefully transferred to a new RNase-free tube. Chloroform (Merck, Hilden, Germany; Cat. No: 102445) was added at a volume of 500 µL, followed by vigorous vortexing for 1 min and incubation for 3 min at room temperature. Samples were centrifuged again at 12,000× *g* for 15 min at 4 °C.

The upper aqueous phase was transferred to a fresh tube, and RNA was precipitated by adding 200 µL of isopropanol (Sigma-Aldrich, St. Louis, MO, USA; Cat. No: I9516). After centrifugation at 12,000× *g* for 10 min at 4 °C, the RNA pellet was washed with 500 µL of 75% ethanol (Merck, Germany; Cat. No: 100983) and centrifuged at 7500× *g* for 10 min. The RNA pellet was air-dried and resuspended in RNase-free DEPC-treated water (Thermo Scientific, Waltham, MA, USA).

RNA concentration and purity were evaluated spectrophotometrically (NanoDrop 2000, Thermo Scientific, Waltham, MA, USA) by measuring absorbance at 260/280 nm. RNA integrity was verified by electrophoresis on a 1.5% agarose gel stained with ethidium bromide.

#### 2.3.2. cDNA Synthesis

To eliminate potential genomic DNA contamination, total RNA samples were treated with DNase I (Thermo Scientific, Waltham, MA, USA; Cat. No: EN0521) following the manufacturer’s protocol. Subsequently, 2–5 µg of purified RNA was reverse-transcribed into complementary DNA (cDNA) using the miScript Reverse Transcription Kit (Qiagen, Hilden, Germany; Cat. No: 218060).

cDNA synthesis was conducted according to the manufacturer’s instructions. The concentration and purity of synthesized cDNA were assessed spectrophotometrically, and samples were stored at −20 °C until further analysis.

#### 2.3.3. Quantitative Real-Time PCR (qRT-PCR)

Gene expression analysis targeted oxidative stress-related genes (SOD1, CAT, GPX1, and GSH), inflammatory cytokines (TNF-α and IL-1β), apoptosis-associated genes (Bax, BCL-2, and Caspase-3), extracellular matrix remodeling markers (MMP-2, MMP-9, and TIMP-1), and bone remodeling markers (RANKL and OPG).

Quantitative real-time PCR was performed using the Rotor-Gene Q real-time PCR system (Qiagen, Hilden, Germany) with SYBR Green detection chemistry (QuantiTect SYBR Green PCR Kit, Qiagen; Cat. No: 204143).

Glyceraldehyde-3-phosphate dehydrogenase (GAPDH) was used as the internal reference gene for normalization. Each reaction was performed in triplicate and contained SYBR Green Master Mix, gene-specific forward and reverse primers, template cDNA, and RNase-free water. Primer specificity was confirmed via melt curve analysis, and amplification efficiencies were verified to fall within acceptable limits.

Relative gene expression levels were calculated using the 2^−ΔΔCt^ method after normalization to GAPDH expression. This approach ensured the accuracy, reproducibility, and robustness of the gene expression analysis.

### 2.4. Statistical Analysis

Statistical analyses were performed using R software (version 4.3.2). The ggplot2 package was used to create all graphical representations to ensure clear and accurate visualization of experimental findings across groups. Statistical significance was determined by a *p*-value below 0.05.

The Kolmogorov–Smirnov test was applied to determine the distribution pattern of the data. This test assesses whether the dataset exhibits a normal (parametric) or non-normal (non-parametric) distribution. The results indicated that most of the data did not meet the assumptions required for the parametric test; therefore, non-parametric statistical methods were used throughout the analysis.

Group comparisons for both ELISA and RT-PCR data were performed using the Kruskal–Wallis test, a nonparametric alternative to ANOVA that is suitable for comparing more than two independent groups when the assumption of normality is not met. When a significant difference was detected via the Kruskal–Wallis test (* *p* < 0.05), Dunn’s post hoc test was applied to determine pairwise differences between specific experimental groups (Control, Force, Force + Levetiracetam, and Force + Valproic Acid).

## 3. Results

### 3.1. Analysis of Oxidative Stress Markers

**Figure 2:**
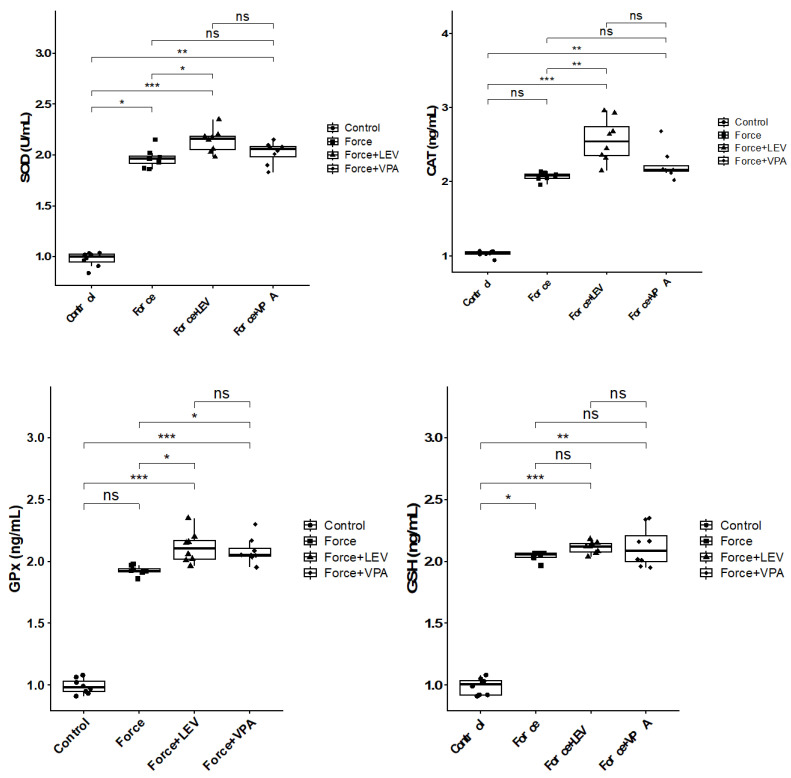
Box plots showing the expression levels of antioxidant parameters according to intergroup comparisons: Superoxide dismutase (SOD), catalase (CAT), glutathione peroxidase (GPx), and reduced glutathione (GSH) levels were compared between groups, and statistical differences were analyzed using the Kruskal–Wallis test and Dunn’s post hoc test. Significance levels: *p* > 0.05 = ns (not significant), *p* < 0.05 (*), *p* < 0.01 (**), and *p* < 0.001 (***).

Alterations in superoxide dismutase (SOD), catalase (CAT), glutathione peroxidase (GPx), and reduced glutathione (GSH) levels in the experimental groups are shown in Figure 2. Significant increases in SOD levels were observed in all force-treated groups compared to the no-force Control group (* *p* < 0.05). The highest SOD values were found in the Force + LEV group, and this increase was statistically significant in comparison to the Force group (* *p* < 0.05). In the Force + VPA group, SOD levels increased compared to the Force group, but this increase was not significant (*p* > 0.05). However, significantly increased SOD expression was observed in the Force + VPA group compared to the Control group (** *p* < 0.01).

There were significant increases in the CAT levels of Force + VPA and Force + LEV groups compared to the Control group (*** *p* < 0.001). CAT activity reached the highest level in the Force + LEV group and was found to be significantly higher than in the Force group (** *p* < 0.01), but it did not show a significant increase compared to the Force + VPA group (*p* > 0.05).

GPx levels of all groups exhibited increases compared to the Control group; however, the increases in the Force + VPA and Force + LEV groups were not statistically significant (*** *p* < 0.001). The GPx level of the Force + LEV group was significantly higher than that of the Force group (* *p* < 0.05), whereas the GPx level of the Force + VPA group was not statistically different from that of the Force group (*p* > 0.05). Similarly, no statistically significant difference was found between the Force + LEV and Force + VPA groups (*p* > 0.05).

GSH levels were found to be significantly higher in all force groups compared to the Control group (* *p* < 0.05). The level of GSH in the Force group was significantly higher compared to the Control group (* *p* < 0.05). GSH levels reached the highest level in the Force + LEV group, and this value was significantly higher than the Force group (** *p* < 0.01). In the Force + VPA group, GSH levels were similar to the Force + LEV group, and both drug groups were higher in comparison to the Control group (* *p* < 0.05). No considerable difference in GSH levels was observed among the Force, Force + VPA, and Force + LEV groups (*p* > 0.05).

### 3.2. Analysis of Pro-Inflammatory Cytokines

**Figure 3:**
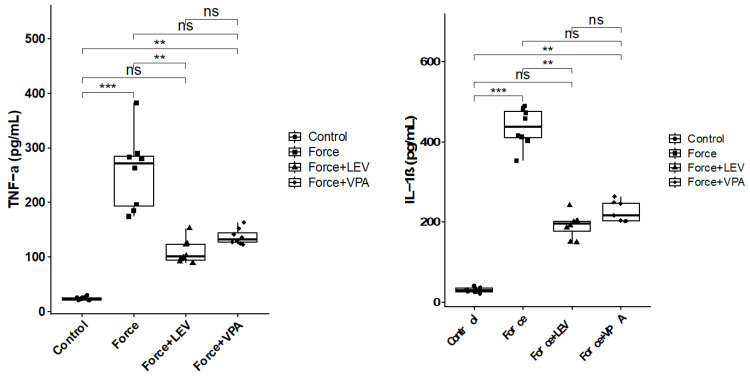
Box plots showing the expression levels of inflammatory parameters according to intergroup comparisons: Tumor necrosis factor-alpha (TNF-α) and interleukin-1 beta (IL-1β) levels were compared between groups, and statistical differences were analyzed using the Kruskal–Wallis test and Dunn’s post hoc test. Significance levels: *p* > 0.05 = ns (not significant), *p* < 0.01 (**), and *p* < 0.001 (***).

As deduced from Figure 3, TNF-α and IL-1β levels were significantly higher in the Force group compared to the Control group (*** *p* < 0.001). TNF-α and IL-1β levels were significantly decreased in the Force + LEV group compared to the Force group (** *p* < 0.01). In the Force + VPA group, TNF-α and IL-1β levels were only slightly decreased compared to the Force group, but this decrease was not significant (*p* > 0.05). Similarly, no significant difference was observed between the Force + LEV and Force + VPA groups (*p* > 0.05).

### 3.3. Analysis of Apoptosis-Related Gene Expressions

**Figure 4:**
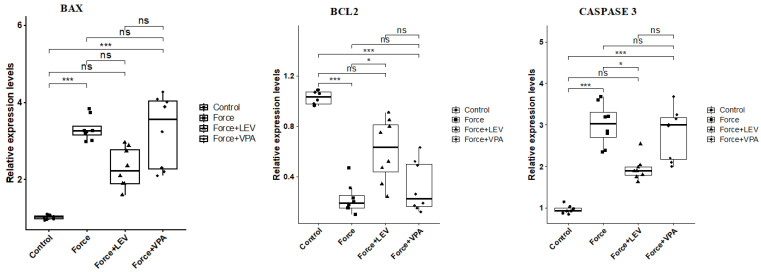
Box plots showing the expression levels of apoptosis-related genes according to intergroup comparisons: BAX, BCL-2, and CASPASE 3 levels were compared between groups, and statistical differences were analyzed using the Kruskal–Wallis test and Dunn’s post hoc test. Significance levels: *p* > 0.05 = ns (not significant), *p* < 0.05 (*), and *p* < 0.001 (***).

As observed in Figure 4, BAX expression was significantly increased in the Force group compared to the Control group (*** *p* < 0.001). BAX levels were significantly decreased in the Force + LEV group compared to the Force group (* *p* < 0.05). In the Force + VPA group, BAX expression was higher compared to the Force group, but the difference was not significant (*p* > 0.05). The Force + VPA group showed significantly higher BAX expression than the Force + LEV group (* *p* < 0.05).

BCL-2 expression was significantly higher in the Control group compared to the force-treated groups (* *p* < 0.05). BCL-2 expression slightly increased in the Force + LEV group compared to the Force and Force + VPA groups, but this increase was not significant (*p* > 0.05).

Caspase-3 expression was significantly increased in the Force group compared to the Control group (*** *p* < 0.001). Caspase-3 levels were significantly decreased in the Force + LEV group compared to the Force group (* *p* < 0.05). In contrast, Caspase-3 expression was significantly higher in the Force + VPA group compared to the Force + LEV group (* *p* < 0.05). No significant difference was found between Force and Force + VPA (*p* > 0.05).

### 3.4. Analysis of Extracellular Matrix Remodeling-Related Biomarkers

**Figure 5:**
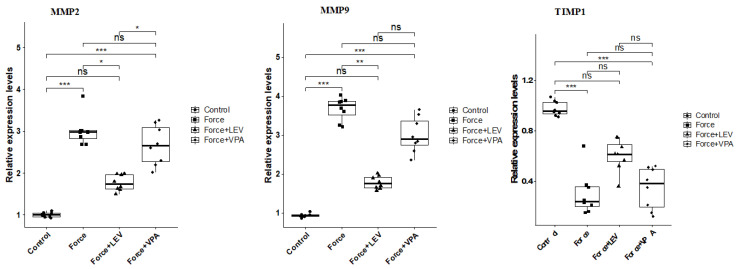
Box plots showing the expression levels of genes associated with extracellular matrix remodeling according to intergroup comparisons: Matrix metalloproteinase-2 (MMP-2), matrix metalloproteinase-9 (MMP-9), and tissue inhibitor of metalloproteinase-1 (TIMP-1) levels were compared between groups, and statistical differences were analyzed using the Kruskal–Wallis test and Dunn’s post hoc test. Significance levels: *p* > 0.05 = ns (not significant), *p* < 0.05 (*), *p* < 0.01 (**), and *p* < 0.001 (***).

MMP-2 expression was significantly increased in the Force group compared to the Control group (*** *p* < 0.001). MMP-2 levels were significantly lower in the Force + LEV group than in the Force group (** *p* < 0.01). In the Force + VPA group, the decrease in MMP-2 expression was more limited than in the Force + LEV group (* *p* < 0.05) (Figure 5). MMP-9 also showed a similar trend to MMP-2 expression. Following force application, MMP-9 levels increased significantly in the Force group compared to the Control group (*** *p* < 0.001). MMP-9 expression decreased significantly in the Force + LEV group compared to the Force group (** *p* < 0.01). In the Force + VPA group, the decrease in MMP-2 expression was more limited (Figure 5).

Changes in TIMP-1 expression levels were in the opposite direction to the other two parameters. The highest TIMP-1 level was found in the Control group, and the lowest was found in the Force group (*** *p* < 0.001). TIMP-1 expression was significantly increased in the Force + LEV group compared to the Force group (* *p* < 0.05), while this increase was more limited in the Force + VPA group (Figure 5).

### 3.5. Analysis of Bone-Remodeling-Related Biomarkers

**Figure 6:**
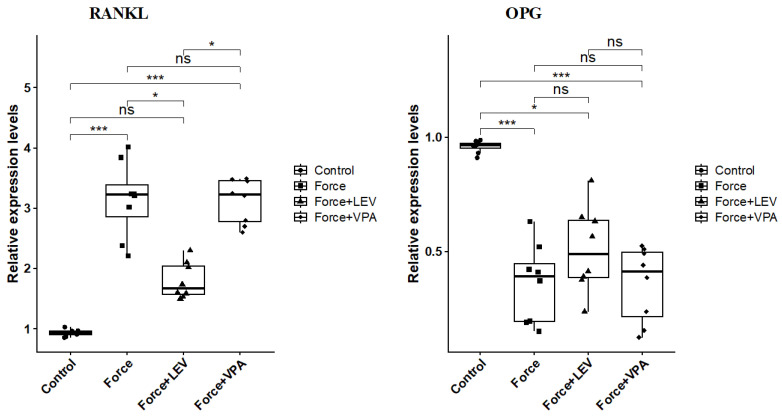
Box plots showing the expression levels of RANKL and OPG genes according to intergroup comparisons: RANKL and OPG levels were compared between groups, and statistical differences were analyzed using the Kruskal–Wallis test and Dunn’s post hoc test. Significance levels: *p* > 0.05 = ns (not significant), *p* < 0.05 (*) and *p* < 0.001 (***).

RANKL expression was significantly increased in the Force group compared to the Control group (*** *p* < 0.001). There was a significant decrease in the RANKL expression of the Force + LEV group compared to the Force group (* *p* < 0.05). RANKL expression reached the highest level in the Force + VPA group. In this group, RANKL expression was significantly higher than the Control group (*** *p* < 0.001) and the Force + LEV group (** *p* < 0.01) (Figure 6).

The results summarized in Figure 6 indicate that the changes in the expression levels of OPG are exactly the opposite of those of RANKL. OPG levels were significantly higher in the Control group than in the other groups (** *p* < 0.01). Although OPG levels in the Force + LEV group were slightly increased compared to the Force group and the Force + VPA group, this difference was not statistically significant (*p* > 0.05).

## 4. Discussion

In dentistry, orthodontic force is mainly used to move teeth, thereby correcting abnormalities in tooth alignment. Orthodontic force refers to a controlled mechanical force that supports tissue remodeling and tooth movement [4]. Under orthodontic force, tooth movement and tissue remodeling are controlled via different cellular and molecular mechanisms, such as inflammation, oxidative stress, and apoptosis [8,9,10]. The normal levels of inflammation, oxidative stress, and apoptosis induced by orthodontic force are essential for bone remodeling; however, their excess levels cause tissue damage and prevent normal bone remodeling and tooth movement [8,11,12,13,33,34]. Therefore, their levels need to be controlled in patients under orthodontic force. This study focused on the effects of antiepileptic drugs (VPA and LEV) on alveolar bone under orthodontic force. Their effects were evaluated through the mechanisms of extracellular matrix (ECM) remodeling, bone remodeling, oxidative stress, inflammation, and apoptosis.

The results of this study revealed that orthodontic force causes an increase in the levels of antioxidant markers (SOD, CAT, GPx, and GSH) when compared to the control. These increases indicate the presence of oxidative stress, namely, reactive oxygen species (ROS) accumulation, induced by orthodontic force. This finding is in good agreement with those of earlier studies, which show that antioxidant enzyme activities increase in cases of oxidative stress caused by orthodontic force [35,36]. The obtained results also uncovered that the levels of antioxidant markers were higher in the LEV group (Force + LEV) compared to the Force group, and the majority of the differences were statistically significant. In the VPA group (Force + VAL), the increases in antioxidant enzyme levels were more limited. These results imply that LEV has a stronger antioxidant response to oxidative stress induced by orthodontic force.

Orthodontic force is also known to cause proinflammatory cell infiltration in periodontal tissue and increases the release of cytokines such as tumor necrosis factor (TNF-α) and interleukin-1β (IL-1β), which play a role in the activation of osteoclasts. These cytokines trigger bone resorption in alveolar bone, paving the way for tooth movement [13,37]. In line with this body of knowledge, the present study revealed that TNF-α and IL-1β levels significantly increased in the Force group compared to the Control group. LEV treatment significantly suppressed the force-induced inflammatory increase, resulting in significant decreases in TNF-α and IL-1β levels. In contrast, VPA administration caused no significant decreases in TNF-α and IL-1β levels. These findings indicate that LEV may inhibit the inflammatory process induced by orthodontic force more potently than VPA.

To date, the antioxidant and/or anti-inflammatory properties of LEV have been investigated in different cell or tissue types; however, there is no study on its antioxidant and inflammatory activities in periodontal tissues, including teeth. For example, Stettner et al. [38] found that LEV treatment reduced oxidative stress in rat Schwann cells in vitro. Kim et al. [39] found that LEV was able to inhibit the development of reactive gliosis and IL-1β expression in brain tissue in an epilepsy model, thereby reducing local inflammation. Yao et al. [40] demonstrated that in experimental animals with ischemic brain injury, LEV reduced TNF-α and IL-1β production in brain tissue and retarded neuronal apoptosis compared to placebo. In summary, the results of the current study and previous studies indicate that LEV possesses strong antioxidant and anti-inflammatory activity.

The results of this study also revealed that orthodontic force increased apoptosis in alveolar bone, as previously reported [41]. The expression levels of the pro-apoptotic BAX and effector caspase-3 genes increased significantly in the Force group compared to the Control group, while the level of the anti-apoptotic BCL-2 decreased significantly. The results also showed that these apoptotic changes were significantly reduced with LEV administration, while VPA failed to prevent these apoptotic changes.

Orthodontic tooth movement is based on ECM remodeling in the periodontal ligament and alveolar bone. When mechanical force is applied, periodontal ligament cells and osteoblasts increase the production of enzymes such as matrix metalloproteinases (MMPs). These enzymes, in turn, degrade ECM components such as collagen and osteoid, thereby facilitating osteoclasts’ access to the bone surface [42,43]. In this study, the Force group was found to have higher levels of MMP-2 and MMP-9 compared to the Control group, while the expression level of TIMP-1 (a natural inhibitor of MMPs) was found to be lower. These results confirm that the applied orthodontic force triggers the enzymatic process that initiates tissue destruction in the alveolar bone. LEV application caused significant decreases in MMP-2 and MMP-9 expression levels and a significant increase in TIMP-1 level compared to the Force group. Unlike LEV, VPA did not cause significant reductions in MMP-2 and MMP-9 levels. All these results indicate that LEV is much more effective than VPA in inhibiting orthodontic force-induced matrix degradation processes.

In the tissue where orthodontic force is applied, inflammation and matrix destruction simultaneously initiate bone remodeling. The balance between osteoclastic bone resorption and osteoblastic bone formation is regulated, particularly through the RANKL (receptor activator ligand) and OPG (osteoprotegerin) axis. Periodontal ligament cells increase RANKL expression and decrease OPG expression in response to mechanical stress, thus stimulating osteoclast activity and resulting in bone resorption [44,45,46]. In this study, RANKL gene expression was significantly increased in the Force group compared to the Control group, while OPG expression was decreased. This suggests that orthodontic force, as expected, creates an environment that enhances osteoclastic activity in the alveolar bone.

This study revealed that LEV and VPA treatments had different effects on the RANKL/OPG balance. RANKL expression increased significantly in the VPA-treated group, even exceeding the RANKL expression level in the Force group. VPA administration did not significantly improve OPG expression. These results are consistent with those of previously published studies, which reveal that VPA increases osteoclastic activity and accelerates bone resorption in humans and animal models by changing the RANKL/OPG balance in favor of RANKL [47,48,49]. For example, in a rat osteoporosis model, it has been clearly shown that VPA administration reduces OPG levels and increases RANKL levels [49]. In the LEV group, RANKL expression significantly decreased compared to the Force group. OPG levels increased slightly in the LEV group compared to the Force group, but this increase was not statistically significant. All these results indicate that orthodontic force application disrupts the RANKL/OPG balance in favor of osteoclast activity, while LEV limits osteoclast activity by decreasing RANKL levels. The previous studies indicated that chronic LEV use may cause negative effects on bone health by decreasing OPG and increasing RANKL [31,50,51]. However, it should be noted that the present study employed short-term LEV administration in an in vivo experimental model. Therefore, the most likely reason for the discrepancy between the results of previous studies and the present study is that LEV has a suppressive effect on the acute inflammatory response. It is known that inflammatory mediators such as IL-1β and prostaglandin E2 can increase RANKL expression in periodontal tissue [52,53,54]. Therefore, LEV may have normalized the RANKL/OPG axis by reducing inflammation in the acute phase. Conversely, with chronic LEV use, especially in the absence of orthodontic stress, the drug’s direct effect on bone cells may have increased RANKL. Another point is that the application period in our study was limited to 6 weeks; longer-term studies may negatively impact LEV’s effects on bone.

Overall, our findings support the hypothesis we proposed at the outset of the study: LEV was more effective than VPA in preventing adverse biological processes, such as oxidative stress, inflammation, apoptosis, and bone destruction, that occur under orthodontic stress.

When orthodontic force is applied to a patient using VPA, the inflammatory and osteoclastic response may be more intense, resulting in more rapid and extensive alveolar bone resorption. This may not only accelerate tooth movement but also increase the risk of the undesirable loss of periodontal support or root resorption. Indeed, the literature has reported that agents that increase bone resorption (e.g., thyroid hormones or parathyroid hormone analogs) can accelerate tooth movement, while agents that inhibit osteoclast activity (e.g., bisphosphonates or OPG) slow tooth movement [25,26]. Based on this information, the higher RANKL levels observed in the VPA group may be associated with more rapid orthodontic tooth movement. However, this may result in uncontrolled bone loss and tissue damage. In contrast, a patient using LEV may have a more controlled tissue response to orthodontic force. By means of LEV’s ability to reduce inflammation and tissue destruction, slower but more physiological tooth movement is likely to occur. While this approach may slightly reduce the rate of movement, it may also better protect periodontal tissue health, reduce pain during treatment, and minimize complications such as root resorption. However, to support this hypothesis, we recommend measuring the effects of LEV and VPA on tooth movement in future animal studies. Furthermore, the results of the current study suggest that physicians consider the adverse effects of certain medications, such as VPA, on bone metabolism when planning orthodontic treatment.

## 5. Conclusions

This study demonstrated that LEV and VPA significantly and differentially impact the biological processes accompanying orthodontic tooth movement. LEV treatment largely offset the molecular and cellular changes induced by orthodontic force and helped maintain tissue integrity, while VPA treatment failed to prevent these changes and even shifted bone metabolism toward osteoclastic activity. These findings provide important evidence regarding how systemically administered medications during orthodontic treatment may affect tissue response and demonstrate that LEV exhibits a more promising profile compared to VPA. All these results suggest that the effects of antiepileptic drugs on bone should be considered when planning orthodontic treatment in individuals using antiepileptic drugs. This study focused on molecular markers, but clinical parameters such as tooth movement volume or bone density were not directly examined. The relationship between the findings obtained at the molecular level and actual orthodontic tooth movement could only be interpreted indirectly. In addition, the relatively short-to-medium study duration, deficiency of histological analysis, the use of only male rats, and the administration of a single drug dose are other limitations of this study. The inclusion of these elements in future studies will allow for stronger implications from both biological and clinical perspectives.

## Data Availability

The data used to support the findings of this study are included within this manuscript.
